# A Mechanistic Overview of Triptolide and Celastrol, Natural Products from *Tripterygium wilfordii* Hook F

**DOI:** 10.3389/fphar.2018.00104

**Published:** 2018-02-14

**Authors:** Shao-Ru Chen, Yan Dai, Jing Zhao, Ligen Lin, Yitao Wang, Ying Wang

**Affiliations:** State Key Laboratory of Quality Research in Chinese Medicine and Institute of Chinese Medical Sciences, University of Macau, Macau, China

**Keywords:** *Tripterygium wilfordii* Hook F, triptolide, celastrol, mechanisms of action, structure-activity-relationship

## Abstract

Triptolide and celastrol are predominantly active natural products isolated from the medicinal plant *Tripterygium wilfordii* Hook F. These compounds exhibit similar pharmacological activities, including anti-cancer, anti-inflammation, anti-obesity, and anti-diabetic activities. Triptolide and celastrol also provide neuroprotection and prevent cardiovascular and metabolic diseases. However, toxicity restricts the further development of triptolide and celastrol. In this review, we comprehensively review therapeutic targets and mechanisms of action, and translational study of triptolide and celastrol. We systemically discuss the structure-activity-relationship of triptolide, celastrol, and their derivatives. Furthermore, we propose the use of structural derivatives, targeted therapy, and combination treatment as possible solutions to reduce toxicity and increase therapeutic window of these potent natural products from *T. wilfordii* Hook F.

## Natural Products Originating From *Tripterygium wilfordii* Hook F Exhibit Distinct Pharmacological Activities

*Tripterygium wilfordii* Hook F (TWHF), also known as Lei Gong Teng (Thunder God Vine), has a long history of improving symptoms of RA (Peterson and Schreiber, 1998; Bao and Dai, 2011; Law et al., 2011). The root bark of TWHF exhibits pharmacological activities against inflammation (Liu et al., 2010; Xue et al., 2012), autoimmune disorders (Chen Y.Z. et al., 2010), fibrosis, atherosclerosis, neurodegeneration (Choi et al., 2010), and kidney diseases (Wan Y.G. et al., 2014). TWHF extracts are used for treating autoimmune and inflammatory diseases in clinical practice (Marks, 2011). In China, the Lei Gong Teng tablet and Lei Gong Teng multiglycoside tablet have been developed for RA treatment since the 1970s (Tao et al., 2001). The chloroform/methanol extract of TWHF attenuates inflammation in patients with Crohn’s disease by inducing the differentiation of Foxp3^+^ T regulatory (Treg) cells and suppressing the serum levels of pro-inflammation cytokines, such as interleukin-10 and TGF-β (Li et al., 2014).

Several classes of bioactive substances have been isolated and characterized from TWHF, including sesquiterpenes, diterpenes (triptolide, tripdiolide, and triptonide), triterpenes (celastrol, pristimerin, and wilforlide A), lignans, glycosides, and alkaloids (Bao and Dai, 2011; Li et al., 2012b; Wang et al., 2013). Triptolide and celastrol are considered as the most active and promising components of TWHF. Thus, their pharmacological activities and mechanisms of action have been extensively investigated in many disease models (**Table 1**).

**Table 1:** Pharmacological activities of triptolide and celastrol *in vivo*.

Disease model	Natural product	Animal model	Experimental detail	Outcome of the study	Reference
RA	Triptolide	Bovine type II collagen-induced RA in male SD rats	30 μg/kg (*s.c.*) for 32 days	Downregulation of RANKL-mediated ERK/AKT signaling pathway	Gong et al., 2017
	Celastrol	Freund’s complete adjuvant-induced RA in C57BL/6 mice	0.5 mg/kg/day (*i.p.*) for 40 days	Suppression the inflammatory activities of neutrophils	Brand et al., 2007; Yuan et al., 2017
Acute promyelocytic leukemia	Triptolide	HL-60 cell implanted xenograft in female NOD/SCID mice	2 or 4 mg/kg (*i.p.*) for 21 days	Activatation of mitogen-activated protein kinase kinase-3/p38 signaling pathway	Pan et al., 2017
	Celastrol	HL-60 cell implanted xenograft in nude mice	2 mg/kg/day (*i.g.*) for 21 days	Mitochondrial-initiated apoptosis	Zhang X. et al., 2016
Colitis	Triptolide	IL-10^-/-^ mice	0.07 mg/kg/other day (*i.p.*) for 8 weeks	Suppression of IL-6/STAT3/SOCS3 signaling pathway	Li et al., 2013
	Celastrol	IL-10^-/-^ mice	2 mg/kg (*i.g.*) for 7 days	Induction of autophage	Zhao J. et al., 2015
	Celastrol	DSS-induced colitis in C57BL/6J mice	1 mg/kg (*i.g.*) for 7 days	Apoptotic cell death	Jia et al., 2015
	Celastrol	Caspase 1^-/-^ mice	1 mg/kg (*i.g.*) for 7 days	Inhibiting activation of NLRP3 inflammasommes	Yu X. et al., 2017
Hepatocellular carcinoma	Triptolide	Huh-7 xenograft in nude mice	Minnelide (prodrug of triptolide) 0.21 mg/kg (*i.p.*) for 7 days	Inhibiting NF-κB activity	Alsaied et al., 2014
	Celastrol	DEN-induced HCC in rat	2, 4, and 8 mg/kg/day (*i.g.*) for 20 weeks	Induction of apoptotic cell death	Chang et al., 2016
Gastric cancer	Triptolide	SC-M1 cell xenograft in SCID mice	0.4 mg/kg (*i.v.*) for 3 weeks	Induction of apoptotic cell death	Li et al., 2012a
	Celastrol	AGS cell xenograft in mice	1–2 mg/kg/day (*i.g.*) for 12 days	Induction of apoptotic cell death	Lee et al., 2014
Osteosarcoma	Triptolide	SAOS2 or U2OS cell xenograft in nude mice	150 nM for 1 to 5 weeks	Induction of apoptotic cell death	Shi et al., 2013; Jiang et al., 2017
	Celastrol	HOS cell xenograft in nude mice	1–2 mg/kg/day (*i.p.*) for 7 days	Apoptotic cell death	Li et al., 2015
Melanoma	Triptolide	B16-F10 cell xenograft in C57BL/6J mice	0.15–0.3 mg/kg daily (*i.g.*) for 14 days	Regulation of inflammatory T cell number and expression of pro-inflammatory cytokines	Liu et al., 2013
	Celastrol	B16 cell xenograft in C57BL/6J mice	1–3 mg/kg (*i.g.*) once a day for 20 days	Inhibition the PI3K/Akt/mTOR signaling pathway	Lee et al., 2012
Pancreatic cancer	Triptolide	SW1990 cell xenograft in BALB/c/nu/nu	0.2–0.4 mg/kg (*i.p.*) for 3 weeks	Supression of HIF-1α through c-MYC-dependent mechanism	Ding et al., 2015
	Celastrol	PANC-1 cell xenograft in nu/nu athymic female mice	3 mg/kg (*i.p.*) for 70 days	Disturbing HSP90-CDC37 interaction	Zhang et al., 2008
Mesothelioma	Triptolide	H2373 or H513 cell xenograft in BALB/c/nu mice	0.42 mg/kg (prodrug of Triptolide, *i.p.*) for 28 days	Suppression of HSP70 expression	Jacobson et al., 2015
Colon cancer	Triptolide	Genotoxic colonic carcinogen- and DSS- induced model in male Crj: CD-1 (ICR) mice	0.1–1 mg/kg (*i.g.*) for 20 weeks	Reducing inflammation, restrict tumor formation and growth	Wang et al., 2009
	Celastrol	Azoxymethane (AOM) and DSS induced colon cancer in C57BL/6J mice	2 mg/kg/day by gavage for 14 weeks	Suppressing inflammatory response and epithelial–mesenchymal transition	Lin et al., 2015
Lung cancer	Triptolide	Orthotopic lung cancer model in nude rats	400 μg/kg intranasal instillation for 8 weeks	Targeting the HA-CD44/RHAMM signaling axis	Song et al., 2017
	Celastrol	A549 or H1975 cell xenograft in Balb/c nude mice	1 or 3 mg/kg/days, or 5 mg/kg, twice/week (*i.p.*) for 3 weeks	Inhibiting CIP2A-Akt pathway	Liu Z. et al., 2014
Neuroinflammation	Triptolide	Middle cerebral artery occlusion in male SD rats	0.2 mg/kg (*i.p.*) at the beginning for 24 h	Inhibition of NF-κB activity	Bai et al., 2016
	Celastrol	Optic nerve crush (ONC) in adult Brown Norway rats	1–5 mg/kg (*i.p.*) for 2 weeks	Activation of TNF-α-mediated cell death	Kyung et al., 2015
Diabetes	Triptolide	High-fat and high-sucrose diet-induced diabetes in Wistar rats	100 μg/kg (*i.g.*) for 8 weeks	Inhibiting inflammation and macrophage infiltration	Ma et al., 2013
	Celastrol	High energy diet and streptozotocin-induced diabetes in male SD rats	1–6 mg/kg (*i.p.*) for 8 weeks	Anti-oxidant activity	Guan et al., 2016
Obesity	Triptolide	Ob/Ob diabetic mice with diabetic nephropathy	25 and 50 μg/kg day for up to 12 weeks	Attenuating albuminuria and renal lesion accompanied with dyslipidaemia and obesity	Gao et al., 2010
	Celastrol	High fat diet-induced obesity in C57BL/6J mice	1–3 mg/kg (*i.g.*) for 2–8 weeks	Increasing sensitivity to leptin through activtion transcription of HSF1-PGC1α	Ma et al., 2015
	Celastrol	Nur77^-/-^ mice injected with LPS+GalN	0.2 or 0.5 mg/kg (*i.p.*) once 12 h before LPS injection	Promoting nuclear receptor 77 translocation from nucleus to mitochondria	Hu M. et al., 2017
	Celastrol	Ob/Ob mice	100 μg/kg (*i.p.*) for 11 days	Increasing leptin sensitivity	Liu J. et al., 2015
Renal damage	Triptolide	Renal ischemia in SD rats	4.17 μmol/day (*i.v.*) for 3 days before renal surgery	Inhibiting proinflammatory cytokines and chemotactic cytokines expression	Fu et al., 2016
	Celastrol	Renal ischemia in rat	4–6 mg/kg (*i.p.*) once 30 min before renal ischemia	Inhibiting NF-κB activation and inflammation	Chu et al., 2014
Cardiovascular disease	Triptolide	Ischemia–reperfusion surgery in Wistar rats	25, 50, and 100 μg/kg 1 h before surgery	Activation of nuclear factor 2/heme oxygenase 1 signaling pathway	Yu et al., 2016
	Celastrol	High-fat/high-cholesterol diet model in apoE^-/-^ mice	1–2 mg/kg (*i.p.*) for 4 weeks	Inhibiting lectin-like oxidized low density lipprotein	Gu et al., 2013
Lung inflammation	Celastrol	Intranasal administration of LPS in male Babl/c mice	1–50 μg/kg (*i.v.*) at the beginning for 12 h	Inhibiting NF-κB signaling pathway	Wang et al., 2014
	Celastrol	NB4 cells-model of differential syndrome in male NOD/SCID mice	300 μg/ml (*i.p.*) for 6 days	Reducing cytokimes, chemokines, and adhesive molecule expression	Xu et al., 2014

## Pharmacological Activities of Triptolide

Triptolide, a diterpenoid triepoxide, was first isolated and characterized from TWHF in Kupchan et al. (1972). Subsequent studies revealed that triptolide exhibit potent pharmacological activities against inflammation, fibrosis, cancer, viral infection, oxidative stress, and osteoporosis (Chugh et al., 2012; Guo et al., 2016; Long et al., 2016). Results from molecular docking and dynamics simulation study suggest that triptolide has similar structures as hormones and thus can also bind to nuclear receptors (Liu X. et al., 2015). Triptolide selectively inhibits the chaperone activity of peroxiredoxin I, an antioxidant enzyme and molecular chaperone that plays essential functions in the development of cancer and inflammation (Zhao Q. et al., 2015). The XBP1 subunit of the transcription factor TFIIH core complex is identified as one of the molecular targets of triptolide, which was critical for the inhibitory activity of triptolide to RNA polymerase II-mediated transcription (Titov et al., 2011). This unique feature is the reason that triptolide is active to most type of diseases in preclinical investigation, such as inflammation and cancer (Han et al., 2012; Yi et al., 2016).

### Anti-inflammation Activities of Triptolide

Triptolide exhibits anti-inflammation activity in T helper cell-mediated immunity especially against RA and inflammatory bowel diseases. It primarily attenuates inflammatory response in RA by inhibiting NF-κB, NF-κB/TNF-α/vascular cell adhesion molecule-1, and TGF-β1/α-smooth muscle/vimentin signaling pathways induced by TNFs and TLR4 (Huang J. et al., 2015; Gong et al., 2017). In RA, triptolide down-regulates the expression levels of myeloid cells-1 and DNAX-associated protein 12 (Fan et al., 2016). In IL-10 deficient mice colitis model, triptolide ameliorates post-surgical intestine inflammation by suppressing the miRNA-155/inositol polyphosphate-5-phosphatease D signaling pathway and producing of inflammatory cytokines (Wu et al., 2013). Triptolide also suppresses IL-6/signal transducers, the activators of transcription (STAT)3/suppressor of the cytokine signaling (SOCS)3 signaling pathway, and promotes apoptotic cell death of lamina propria mononuclear cells (Li et al., 2013).

Triptolide suppresses abnormally activated innate immune response and attenuates LPS-induced acute lung injury by decreasing leukocyte numbers, myeloperoxidase activity, and secretion of pro-inflammatory cytokines such as TNF-α, IL-1β, and IL-6 (Wang et al., 2014). The suppression of prostaglandin E2 receptor 2 signaling pathway leads to the inhibitory activity of triptolide on LPS-induced inflammation and expression of inducible nitric oxide synthase in microglia (Zhang et al., 2015). Treatment with triptolide prevents kidney damage by reducing the expression level of malondialdehyde and increasing superoxide dismutase activity by modulating the NF-κB signaling pathway in rats (Zhou Y. et al., 2016). The formation of the NLRP3 inflammasomes is abrogated by triptolide in transverse aortic constriction in mice (Li et al., 2017). Triptolide diminishes neuroinflammation through the downregulation of p38 MAPK and NF-κB signaling pathways in rat models of depression or middle cerebral artery occlusion, respectively (Wan B. et al., 2014; Xu et al., 2015; Bai et al., 2016; Zhang B. et al., 2016; Hu G. et al., 2017; Hu X. et al., 2017).

### Anticancer Activities of Triptolide

Triptolide suppresses the proliferation and promotes apoptotic cell death in various cancers, especially hard-to-treat types, including prostate and pancreatic cancers. Prostate and pancreatic cancers are the third and fourth leading causes of cancer death in western countries, respectively (Isharwal et al., 2015). Treatment with triptolide induces the nuclear accumulation of p53 and apoptotic cell death in primary cultures of human prostatic epithelial cells (Kiviharju et al., 2002). The growth of prostate cancer xenograft in nude mice is inhibited after triptolide decreases the expression levels of SUMO-specific protease 1 and androgen receptor (Huang et al., 2012). Furthermore, the sensitivity of gemcitabine-resistant pancreatic cancer cells to cisplatin treatment is enhanced by triptolide through activation of mitochondria-initiated cell death pathway and suppression of HSP27 expression (Zhu W. et al., 2012).

The generally recognized molecular target of triptolide is the XBP1 subunit of transcription factor TFIIH (Titov et al., 2011). Triptolide covalently binds to the Cys342 of XBP1 by the 12,13-epoxide group (He et al., 2015), and thus inhibits transcription and nucleotide excision repair activity of RNA polymerase II in an ATP-dependent manner (Pan, 2010; Titov et al., 2011). In addition, triptolide induces phosphorylation of RPB1 subunit of RNA polymerase II on Ser1878 by activating cyclin-dependent kinase (CDK)7, which promotes degradation of RPB1 and subsequently induced cancer cell death (Manzo et al., 2012).

Developing MDR is one of the biggest challenges for cancer therapy (Joshi et al., 2017). Triptolide suppresses the expression of MDR protein, and induces apoptotic cell death of drug-sensitive parental KB cells and multidrug-resistant KB-7D and KB-tax cells (Chen Y.W. et al., 2010). The inhibitory activity on MDR cell lines is mediated through the suppression of overall transcription mediated by RNA polymerase II in a CDK7-dependent manner. In addition, triptolide alters the activity of *p*-glycoprotein drug efflux and mRNA level of MDR genes (Yi et al., 2016).

## Pharmacological Activities of Celastrol

### Pharmacological Activities of Celastrol Mediated through HSP90

Celastrol was first reported to inhibit HSP90 by promoting the nuclear translocation of HSF1, a transcription factor regulating HSP genes by binding to the heat shock elements in yeast and mammalian cells (Trott et al., 2008; Akerfelt et al., 2010; Der Sarkissian et al., 2014). Thus celastrol was defined as a HSP90 inhibitor.

Classical HSP90 inhibitors suppress the ATPase activity of HSP90 (Fontaine et al., 2015). Celastrol induces the degradation of HSP90 and its client proteins without interfering the bonds between ATP and HSP90 in pancreatic cancers and NSCLC in micromolar concentration (Zhang et al., 2008; Fan et al., 2014). Treatment with celastrol also induces the dephosphorylation and degradation of HSP90/CDC37 client protein kinases, including Raf family proteins, AKT, MEK1/2, CDK4, and epidermal growth factor receptor (EGFR) in HCC cells, leading to the inhibition of proliferation and induction of apoptotic cell death (Wei et al., 2014). Suppressing the expression of HSP90 by celastrol also sensitizes glioblastoma cells to celastrol treatment (Boridy et al., 2014).

HSP90 activity can be modulated through celastrol treatment. Celastrol restores the disrupted association between HSP90 and its co-chaperone CDC37/HSP90-HSP70 complex, and thus reduces HSP90-mediated degradation of glucocerebrosidase (GCase) (Yang et al., 2014; Van Rossum and Holsopple, 2016). The resulting increased quantity and catalytic activity of GCase compensates for glucosidase mutation, which is the primary cause of Gaucher disease (Irun et al., 2013; Yang et al., 2014). Treatment with celastrol also induces expression of HSP72, phosphorylation, and nucleus accumulation of HSF1, which abrogates proteasome activity in dexamethasone-induced atrophy (Gwag et al., 2013).

### Anticancer Activities of Celastrol Independent of HSP90

Celastrol effectively inhibits the growth of melanoma xenograft in mouse by triggering ROS-mediated caspase-dependent and caspase-independent apoptotic cell death, and inactivating the PI3K/AKT signaling pathway with the dosage of 1 mg/kg (Lee et al., 2012). Treatment with celastrol also suppresses the proliferation of osteosarcoma and bladder cancer cells via ROS/c-JNK and mitochondrial apoptotic pathways, and induces apoptosis and autophagy both *in vivo* and *in vitro* (Li et al., 2015; Yu et al., 2015).

Celastrol treatment initiates programmed cell death by activating glycogen synthase kinase-3β (Feng et al., 2013). The treatment induces cell cycle arrest, activation of caspase 3/7, and apoptosis in c-Met-deficient Huh7 cells, Bel 7402 cells, and diethylnitrosamine-induced liver cancer in rats (Chang et al., 2016). It inhibits the viability of HepG2 cells by suppressing the TLR4-NF-κB signaling pathway when used alone (Shen et al., 2016), and suppresses EGFR expression when combined with lapatinib (Yan et al., 2014). Celastrol also inhibits cancer growth by activating TNF-α-induced NF-κB signaling pathway (Kang et al., 2013), inhibiting the mTOR/ribosomal protein S6 kinase/eIF4E/AKT and ERK signaling pathway, and down-regulating hypoxia-inducible factor-1α (HIF-1α) (Ma et al., 2014). Celastrol inhibits the growth of MCF-7 breast cancer cells through AMPK (Kim et al., 2013), and induces apoptosis in HT-29 colon adenocarcinoma cells through the canonical WNT/β-catenin pathway (Lu et al., 2012). Furthermore, celastrol suppresses the proliferation and invasion of ulcerative colitis-related colorectal cancer and NSCLC by inhibiting epithelial–mesenchymal transition, increasing E-cadherin expression, down-regulating N-cadherin, vimentin, and snail (Lin et al., 2015; Lo Iacono et al., 2015).

Treatment with celastrol inhibits the proliferation, migration, and invasion of chondrosarcomas cells by suppressing the inhibitor of the protein phosphatase 2A-Akt signaling pathway *in vivo* (Liu Z. et al., 2014). Celastrol rapidly reduces the expression level of c-MYC protein and stimulates an energy crisis by depleting ATP, inducing accumulation of neutral lipid, and activating AMPK, thereby ultimately induces cell cycle arrest and apoptotic cell death in human cancer cells (Wang et al., 2015). In addition, celastrol inhibits the growth of head and neck cancer cells by activating C/EBP-homologous protein and XBP1s, which increase the transcription of ER stress target genes and subsequently induces ER stress (Fribley et al., 2015).

### Anti-inflammation Activities of Celastrol Independent of HSP90

Treatment with celastrol reduces inflammation in several disease models independent of HSP90. Celastrol significantly suppresses inflammation by reducing the secretion of IL-1β and IL-18, and inactivating the NLRP3 inflammasomes and caspase-1 in LPS/ATP-primed macrophage cells (Xin et al., 2017; Yu X. et al., 2017). Celastrol ameliorates colitis in IL-10-deficient mice by reducing colon myeloperoxidase concentration and inhibiting colonic pro-inflammatory cytokines via PI3K/Akt/mTOR signaling pathway (Zhao J. et al., 2015). It also ameliorates dextran sulfate sodium-induced colitis in caspase-1^-/-^ mice by inhibiting the activation of NLRP3 inflammasomes and the subsequent secretion of IL-1β (Yu X. et al., 2017). Celastrol potentiates mitochondrial damage and inflammation in palmitate-induced insulin resistance in C3A hepatocytes (Abu Bakar et al., 2017). Treatment with celastrol also inhibits acute liver inflammation through the Nur77-dependent pathway and activation of autophagy in LPS and D-galactosamine-induced inflammation in mice (Greenhill, 2015). Although it inhibits the differentiation of Th17 cells, celastrol enhances the production of Treg cells by restricting the activation of STAT3 and its downstream target genes. In adjuvant arthritis rats, these processes suppress joint inflammation (Astry et al., 2015).

### Activities of Celastrol in Metabolic Diseases

Metabolic diseases are often associated with over-activated inflammatory response, for example obesity and diabetes (Straub, 2017). Delaminating pathogenic factors of the disease progression lead to reduced inflammation. Celastrol inhibits obesity and metabolic dysfunction by increasing energy expenditure, activation of brown adipose tissue, and expression of mitochondrial genes *in vivo*. Treatment with celastrol activates the transcription of HSF1 and expression of peroxisome proliferative activated receptor coactivator-1α in mouse adipose tissues and muscle (Ma et al., 2015), promoting thermogenesis and the remodeling of white adipose tissues. In addition, celastrol inhibits high fat diet-induced obesity in Nur77^-/-^ mice by activating autophagy and promoting the interaction between Nur77 and TRAF2 (Hu M. et al., 2017). Celastrol treatment reduces body weight gain and food intake in both high fat-fed obese in diabetes (db/db) and leptin-deficient (ob/ob) mice by potentiating STAT3-dependent leptin signaling and inhibiting ER stress (Liu J. et al., 2015).

## Translational Development of Triptolide and Celastrol

Despite the various activities offered, clinical application of triptolide and celastrol remains limited due to their narrow therapeutic window and poor solubility (Wang et al., 2016). The major toxicity of triptolide includes hepatic and cardiac toxicity. Triptolide induces hepatotoxicity by suppressing the transcription of four major cytochromes P450 (CYP) isoforms, namely CYP3A, CYP2C9, CYP2C19, and CYP2E1, thus decreasing the substrate affinities and activities of these CYP enzymes, which are vital to normal metabolism regulation (Lu et al., 2017). Decrease in CYP enzymes antagonizes the activity of other drugs metabolized through these CYP isoforms (Lu et al., 2017). Treatment with triptolide leads to cardiac cytotoxicity through induction of oxidative stress, mitochondrial dysfunction, and apoptotic damage regulated by the mitochondria-mediated apoptotic signaling pathway in cardiomyocytes (Zhou et al., 2014). Considerable effort has been exerted for the reduction of toxicity of triptolide and celastrol, including combination therapy, nanoparticle coating for enhancement of water solubility and targeted delivery, and development of water-soluble analogs of triptolide and celastrol. The structural basis of the toxicity of TWHF compounds can be determined through in-depth understanding of the relationship between structures and activities of their functional analogs.

### Combination Treatment for Toxicity Reduction

Lowering the dosage used of TWHF compounds by combining them with agent(s) effectively reduces their cytotoxicity and related adverse effects. Combination treatment offer new opportunities for the translational development of triptolide and celastrol. Active components from the same herb can synergize with other compounds isolated from the same herb (Zhou X. et al., 2016). For example, combination treatment of triptolide and celastrol synergistically inhibits cell growth, induces cell cycle arrest at G2/M phase and apoptosis, and increases intracellular ROS accumulation in many types of cancer cells, including H1299 and H157 lung cancer cells (Jiang et al., 2015).

Triptolide has been used for the reduction of resistance against chemotherapeutic agents. Low doses of triptolide reverses resistance to cytarabine and doxorubicin in acute lymphoblastic leukemia cell line NALM-6/R and primary cells isolated from patients with relapsed or refractory acute lymphoblastic leukemia in the mouse xenograft model (Zhao et al., 2016a). The combination treatment of triptolide and oxaliplatin significantly inhibits the proliferation of colon cancer cell lines by regulating the Wnt/β-catenin pathway in nanomolar concentration (Liu Y. et al., 2014). Triptolide together with hydroxycamptothecin exhibits broad-spectrum anticancer activities by regulating MAPK and Akt signaling pathways (Meng et al., 2015). Combination treatment with farnesoid X receptor activator GW4064 relieves triptolide-induced hepatic toxicity (Jin et al., 2015). The treatment reduces incidence of spontaneous lung cancer from 70 to 10% by potentiating the NF-κB signaling pathway-mediated production of proinflammatory cytokines *in vivo* when used in a low dose (1 mg/kg body weight) and in combination with aspirin (Zheng et al., 2017).

The combined treatment of celastrol and histone deacetylases inhibitor suberoylanilide hydroxamic acid simultaneously activate the NF-κB and E-cadherin signaling pathway, thus substantially inhibit growth of human cancer cells *in vitro* and *in vivo* (Zheng et al., 2014). When combined with ABT-737, a BH3 mimetic inhibitor of Bcl-xL, Bcl-2, and Bcl-w, celastrol synergistically suppresses the proliferation of HCC cells and induces apoptotic cell death (Zhu H. et al., 2012). The combination treatment with triptolide and celastrol exhibits synergistic anticancer activity in H1299 human NSCLC cells and H157 human oral carcinoma xenografts by inhibiting HSP90 activity and reducing ROS level (Jiang et al., 2015).

### Nanoparticle Coating to Enhance Solubility and Organ Targeting

Nanoparticle is used as drug delivery system to improve therapeutic efficacy by increasing solubility and organ targeting through electrostatic interactions or receptor and/or membrane binding (Zhou et al., 2017).

Follicle-stimulating hormone (FSH)-β-peptide modified nanoparticle is used to increase water solubility and reduce cytotoxicity of triptolide in ovarian cancer mouse model (Chen X.Y. et al., 2015). Administration of FSH-β-peptide nanoparticle containing triptolide suppresses the serum level of antimullerian hormone, reduces prominent ovarian fibrosis, vacuolar changes, and follicle numbers (Chen X.Y. et al., 2015). A nanoparticle formed with PF-A299-585 (amino acid 299–585) peptide fragment of human serum albumin increases the kidney targeting property of triptolide, and exhibits comparable anti-inflammatory activity with less cytotoxicity in LPS-stimulated Madin-Darby canine kidney cells and in membranous nephropathic rodent model (Yuan et al., 2015). Triptolide packed with the reduction-sensitive lipid-polymer hybrid nanoparticles inhibits proliferation of human oral cavity squamous cell carcinoma cells more effectively with a low combination index with doxorubicin and reduced cytotoxicity (Wu et al., 2017). Coating with pH-sensitive floating nanoparticles allows specific uptake of triptolide by liver cancer cells, results in improved efficacy as well as reduced cytotoxicity compared with triptolide alone *in vivo* and *in vitro* (Ling et al., 2014). Disodium phosphonooxymethyl nanoparticle extends the shelf life of triptolide, reduces the dosage required to inhibit growth of human ovarian cancer xenograft compared with that of triptolide (Patil et al., 2015).

PEGylated distearoyl phosphatidylcholine liposomes packed with celastrol exhibits higher efficiency in inducing apoptotic cell death in prostate cancer cells, compared with celastrol dissolved in dimethyl sulfoxide (Wolfram et al., 2014). This formula prolongs the blood circulation time of celastrol, increasing bioavailability and reducing the frequency of dosing (Wolfram et al., 2014). Celastrol nanoparticles significantly inhibits suture-induced corneal neovascularization by reducing macrophage infiltration and decreasing the expression of vascular endothelial growth factor and matrix metalloproteinase 9 in rat cornea (Li et al., 2012c; Sanna et al., 2015). Mesoporous silica nanoparticle and axitinib in PEGylate lipid bilayers increases the inhibitory activity of celastrol on angiogenesis and mitochondrial function, and enhances its anticancer activity in SCC-7, BT-474, and SH-SY5Y cells (Choi et al., 2016). Glucose-functionalized mesoporous silica nanoparticles significantly enhances tissue targeting and inhibitory activity of celastrol toward Hela and A549 cancer cells (Niemela et al., 2015). Celastrol-albumin nanoparticles exhibits tissue targeting property to mesangial cells, and attenuates proteinuria, inflammation, and glomerular hypercellularity in mesangial cell-mediated glomerulonephritis (Guo et al., 2017). Celastrol-albumin nanoparticles reduces the accumulation of free celastrol in off-target organs and tissues, thus successfully reducing the systemic toxicity of celastrol (Guo et al., 2017).

### Development of Water-Soluble Analogs of Triptolide and Celastrol

Several water-soluble analogs of triptolide and celastrol were synthesized and evaluated in laboratory animal models and clinical trials. PG490-88, as a water-soluble succinate salt analog of triptolide, more specifically and effectively blocks pulmonary fibrosis in intratracheal bleomycin mouse model than triptolide (Krishna et al., 2001). PG490-88 also inhibits the growth of cancer-derived primary cultures of human prostatic epithelial cells in a p53-dependent manner (Kiviharju et al., 2002). MRx 102, a triptolide derivative with C-14-hydroxyl modification of amine ester group, differentially regulates the expression of retinoid X receptor-α (RXRα) but not the level of full length of RXRα, and inhibits cancer cell growth through the inhibition of AKT signaling pathway (Wang et al., 2017).

(5*R*)-5-hydroxytriptolide (LLDT-8) is a triptolide analog with a favorable safety profile, the efficacy of which will be examined for the treatment of RA and cancer in phase II clinical trials (Wang L. et al., 2012; Qi et al., 2017). LLDT-8 maintains the immune suppressive activity of triptolide (Zhang et al., 2017). Treatment with LLDT-8 ameliorates anti-GBM glomerulonephritis by regulating the Fcγ receptor signaling (Qi et al., 2017). LLDT-8 exhibits anti-inflammation activity and inhibits transcription in a similar fashion as triptolide (Chen et al., 2016). However, testis toxicity is associated with long-term LLDT-8 treatment due to its upregulation of TGF-β activated kinase 1 (Yu C. et al., 2017).

Minnelide, a phosphonooxymethytriptolide disodium salt, was synthesized by reacting triptolide with acetic anhydride in dimethyl sulfoxide at room temperature for 5 days (Chugh et al., 2012). Minnelide inhibits the growth of multiple cancers in pre-clinical studies (Chugh et al., 2012; Jacobson et al., 2015; Oliveira et al., 2015; Arora et al., 2017; Isharwal et al., 2017), for example, colon cancer and metastasis to liver (Oliveira et al., 2015). The growth of pancreatic cancer in KRas and TP53 mutant mouse model (KRas^G12D^; Trp53^R172H^; Pdx-1Cre) is also attenuated by minnelide (Chugh et al., 2012). No overt signs of toxicity is observed during more than 1 year’s treatment of minnelide in athymic nude mice bearing human pancreatic cancer xenograft (Chugh et al., 2012); thus the therapeutic window of minnelide is greatly enhanced than that of triptolide. The efficacy of minnelide in patients with refractory pancreatic cancer is currently evaluated in phase II clinical trial (NCT03117920).

Few C6-indole modified water soluble analogs of celastrol were synthesized. NST001A, a sodium salt of celastrol, inhibits the growth of human colon cancer cell-Colo 205 colon cells *in vitro* and *in vivo* (Tang et al., 2014). Two celastrol derivatives (NST001 and NST001B) also exhibits enhanced potency against the growth of HCC cells than celastrol (Tang et al., 2014). CEL20 disrupts the interaction of HSP90-CDC37 more efficiently than celastrol in A549, MCF7, and pancreatic Panc-1 human cancer cell lines (Jiang et al., 2016).

### Structure-Activity-Relationship of Compounds Isolated from TWHF

The structural-activity-relationship of major compounds isolated from TWHF against key signaling pathways regulating inflammation have been studied by many groups, aiming to evaluate SAR for the future modifications of TWHF compounds.

*Tripterygium wilfordii* Hook F compounds including triptolide with epoxide are attacked by a well-positioned -SH, which is one of the determinant factors for their pharmacological activities (He et al., 2015). Triptolide binds to the cysteine residues of target proteins through covalent bond; and thus modifies the properties and activities of the target proteins. However, no epoxide moiety is observed on other TWHF compounds such as withaferin A and celastrol, although they exhibits the same activities as triptolide (Zhao Q. et al., 2015). In withaferin A and celastrol, 1,4-dipolar structure constructed by the carbonyl and adjacent double carbon-carbon bond that binds to the cysteine residues of the target proteins. The epoxide and 1,4-dipolar structure are both electrophilic groups that can be attacked by -SH via ring-opening and Michael reactions, respectively. Therefore, the covalent bond between the electrophilic structure of TWHF compounds and cysteine residues on target proteins could be considered as determinant factor for the SAR of TWHF compounds.

The difference in the Michael addition and ring-opening reactions of celastrol, pristimerin, and triptolide may have led to the huge difference among the biological activities of these TWHF compounds. TWHF compounds with the electrophilic structure, including triptolide, celastrol, and pristimerin exhibit similar inhibitory activities against NF-κB, STAT3, and SMAD2/3 signaling pathways (**Table 2**). Comparatively, the potency of celastrol and pristimerin against key signaling pathways regulating inflammation is lower than that of triptolide (**Table 2**). Especially that the concentration of pristimerin required to suppresses NF-κB activation already reduces cell viability, suggesting that the immune suppressive activity of pristimerin is due to the inhibition of cell growth. This is in line with other reports that pristimerin minimally inhibits the proliferation of cancer cells (Huang S. et al., 2015; Zhao et al., 2016b).

**Table 2:** Activities of major compounds isolated from THWF on different signaling pathways.

Nature product	Activity (EC_50_^a^, detection method)	Cell type and viability at EC_50_ (detection method)	Reference
Triptolide	Inhibit TRAIL-induced NF-κB at 20 ng/ml (reporter assay)	Lung cancer A549 and NCI-H1299 cell lines, 75–90% (MTT assay)	Lee et al., 1999, 2002
Triptolide	Inhibit IL-6-stimulated STAT3 at 30 nM (reporter assay)	Colon cancer cell line SW480 cells, 10% (MTT assay)	Wang et al., 2009
Triptolide	Inhibit TGF-β1-activated SMAD2/3 at 10 nM (Western blot analysis)	Airway smooth muscle cells, 70% (MTT assay)	Chen M. et al., 2015
Celastrol	Inhibit TGF-β1-stimulated NF-κB at 1000 nM (Western blot analysis)	Squamous cell carcinoma 228 cell, 90% (MTT assay)	Freudlsperger et al., 2013
Celastrol	Inhibit TNF-α-stimulated NF-κB at 3000 nM (reporter assay)	Human embryonic kidney subclone A293 cells, 80% (MTT assay)	Sethi et al., 2007
Celastrol	Inhibit IL-6-stimulated STAT3 at 1000 nM (Western blot analysis)	Multiple myeloma U266 cells, 90% (MTT assay)	Kannaiyan et al., 2011
Pristimerin	Inhibit NF-κB p65/DNA binding activity at 300–400 nM (ELISA assay)	BXPC-3, PNCA-1, and AsPC-1 pancreatic cancer cells, 50% (cell counting)	Wang Y. et al., 2012
Pristimerin	Inhibit NF-κB p65 protein expression at 600 nM (Western blot analysis)	PNCA-1 cells, 25% (MTS assay)	Deeb et al., 2015
Pristimerin	Inhibit LPS-stimulated NF-κB activation through TLR4 at 500 nM (p65/DNA binding assay)	Cellosaurus BV2 cells, 100% (MTT assay)	Hui et al., 2018

*^*a*^50% inhibition of efficacy to signaling pathway to untreated control.*

**FIGURE 1:**
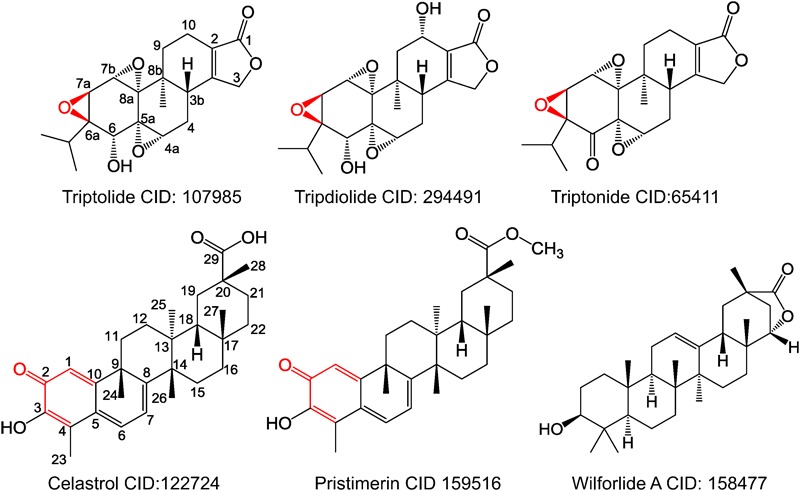
Chemical structures of compounds isolated from *Tripterygium wilfordii* Hook F. Red color labeled moieties essential to maintain biological activities.

Inspired by the epoxide’s effect on protein proposed by Zhao Q. et al. (2015), the epoxide moieties on the C-6a and C-7a positions of triptolide could act as acceptors for cysteine (**Figure 1**). Triptolide directly binds to cysteine via the ring-opening interaction between epoxide and -SH moiety of cysteine, which could lead to the dissociation of these proteins to their downstream target elements. Instead of epoxide, the 1,4-dipolar structure constructed by the carbonyl on C-2 and its adjacent alkenyl on celastrol and pristimerin acts as an acceptor of cysteine (**Figure 1**). This structure can combine with -SH by the Michael reaction. The activities of the Michael addition and ring-opening reaction might have caused the differences among the pharmacological activities of celastrol, pristimerin, and triptolide. Wilforlide A without epoxide or 1,4-dipolar structure is inactive to all these signaling pathways, and only exhibits marginal potency against inflammation in RA rats (Xue et al., 2010). Optimized derivatives with reduced toxicity may be used for further translational development.

## Summary

On the basis of the pharmacological activities of compounds isolated from THWF, especially triptolide and celastrol, we conclude that the systemic evaluation of the *in vivo* activities of these compounds is still needed. Targeted delivery systems, structure modifications, and the mechanisms of action of these compounds are essential in the design of novel derivatives with reduced cytotoxicity, improved efficacy, and increased therapeutic index.

## Author Contributions

S-RC, YtW, and YW mainly drafted the work critical for important intellectual content; YD, JZ, and LL finished the SAR discussion; all authors approved the version to be published.

## Conflict of Interest Statement

The authors declare that the research was conducted in the absence of any commercial or financial relationships that could be construed as a potential conflict of interest.

## Abbreviations

AMPK: AMP-activated protein kinase
ATP: adenosine triphosphate
BMP: bone morphogenetic protein
CDC: cell division cycle
CDK: cyclin dependent kinase
CYP: cytochrome protein
DEN: diethylnitrosamine
ER: endoplasmic reticulum
GCase: glucocerebrosidase
HCC: hepatocellular carcinoma
HSF: heat shock factor
HSP: heat shock protein
IFN: interferon
IL: interleukin
LPS: lipopolysaccharide
MDR: multi-drug resistance
mTOR: mammalian target of rapamycin
NF: nuclear factor
NLRP3: NLR family pyrin domain containing 3
NSCLC: non-small cell lung carcinoma
RA: rheumatoid arthritis
ROS: reactive oxygen species
RXRα: retinoid X receptor-α
SHIP: inositol polyphosphate-5-phosphatease
STAT: signal transducer and activator of transcription
TGF: transforming growth factor
TLR: toll-like receptor
TNF: tumor necrosis factor
Treg: T regulatory
TWHF: *Tripterygium wilfordii* Hook F
XBP1: X box binding protein 1
